# Prevalence of Unhealthy Lifestyles and Its Correlates Among College-Going Students in Rishikesh, India: A Cross-Sectional Study

**DOI:** 10.7759/cureus.64713

**Published:** 2024-07-17

**Authors:** Radhika Yadav, Meenakshi Khapre, Diksha D, Anjali M

**Affiliations:** 1 Public Health, All India Institute of Medical Sciences, Rishikesh, Rishikesh, IND; 2 Community and Family Medicine, All India Institute of Medical Sciences, Rishikesh, Rishikesh, IND; 3 Community Medicine, Dr Moopen's Medical College, Wayanad, IND

**Keywords:** dietary habit, physical activity, attitudes, health knowledge, chronic disease, adolescent, lifestyle

## Abstract

Introduction

Non-communicable diseases (NCDs) have a significant impact on health concerns. The transition from school to college coincides with various stressors, affecting student health. The objective was to assess the prevalence of unhealthy lifestyles among college-going students and examine the association of composite lifestyle scores with selected demographic variables.

Methods

All undergraduate colleges of Rishikesh were included in the study. Cluster sampling with a population proportionate to size was used to select the participants. A structured questionnaire was administered using Google Forms or pen and paper. Index values for each lifestyle activity were added together to get a lifestyle composite score. An unhealthy lifestyle was considered with a score below 40. Association with risk factors was analyzed using a chi-square test and logistic regression.

Results

Among 742 participants, 166 (22.4%) lived an unhealthy lifestyle with a lifestyle score of <40. The majority of participants were either underweight or above normal weight. An unhealthy lifestyle was prevalent among fourth-year professional students (7; 58.5%), medical (33; 32%), dental (27; 34.6%), hosteller (79; 36%), and BSc Nursing (21; 56.8%) students. BSc Nursing course (AOR: 11.09; 95% CI: 1.17-104.74) and favorable attitude (AOR: 0.74; 95 % CI: 0.59- 0.93) were significant correlates of unhealthy lifestyles.

Conclusion

The current study indicates that nearly one-fourth of college students have unhealthy lifestyles. Four factors, i.e., health science stream, advancing professional year, hosteller, and unfavorable attitude toward health, were significantly associated with unhealthy lifestyles after adjusting for covariates.

## Introduction

Lifestyle is devoted to the specifications of inhabitants of a region at a particular time and place [1]. A healthy lifestyle is a way of living that helps the individual enjoy life across more dimensions and features. A healthy lifestyle reduces the risk of severe illness and increases life expectancy.

According to WHO, non-communicable diseases (NCDs) contribute to around 38 million (68%) deaths globally and about 5.87 million (60%) deaths in India [2]. Two-thirds of deaths in Indian adults are co-related with lifestyle practices. Indian Council of Medical Research (ICMR) data reflects that many Indians are physically inactive, with more than six million people dying early from tobacco-related diseases [3]. There are approximately 120 million smokers in India, and 41.7 % of adults are engaged in alcoholism [4]. If such risky behavior is continued, chronic disease burden will negatively impact the country's population health, health system, and economy. Chronic diseases commonly develop in middle age after prolonged exposure to an unhealthy lifestyle involving tobacco use, a lack of physical activity, and a diet loaded with saturated fats, sugars, and salt from young adulthood [5].

An unhealthy life is modifiable and generally constituted during young adulthood. College students are notably engaged in unhealthy lifestyle practices [6]. The transition from school to college is generally co-related, with a significant blend of stressors affecting student health [7]. This midway period is crucial for developing lifelong healthy attitudes and practices and avoiding the biological precursors of chronic disease in life and throughout life. Therefore, preventing non-communicable diseases is important for young people now and in the future. Previous studies in the university population have proved the association between sociodemographic risk factors and unhealthy lifestyles [8-17] and the impact of clustering risk factors [18,19].

The findings from this study can provide information on the prevalence of unhealthy lifestyles and risk factors among college students who represent the future vitality of the nation. It would also enable health professionals to realize and assess how behaviors cluster together so that they can devise a focused group intervention strategy.

Objectives

This study was conducted from May 2020 to May 2021 among the college-going students of Rishikesh to find the prevalence of unhealthy lifestyles in terms of physical activity, dietary habits, sleep, alcohol, tobacco, and stress using a composite lifestyle index tool [20] and estimate the association of lifestyle score with selected sociodemographic variables, knowledge, attitude, and presence of chronic disease.

## Materials and methods

Study setting and design

A cross-sectional study was conducted in seven colleges in Rishikesh, India, delivering courses like medicine, nursing, dentistry, management, engineering, science, arts, and commerce. Rishikesh is a city that is located in Uttarakhand state, India. The urban area of Rishikesh is divided into seven towns with a population of 7,000 to 70,000. Rishikesh has a total of seven colleges, belonging to different streams like medical, nursing, dental, management, engineering, science, arts, and commerce, with a cumulative strength of students of approximately 3563. The study was conducted from May 2020 to May 2021.

Study participants

All students of the age range 18-24 years (from first to final year) of different colleges of Rishikesh and students present on the day of the visit physically/online mode were invited to participate in the study. Students who refused to give consent were excluded.

Sample size and sampling

With the assumption of getting a maximum sample size prevalence of 50 % was considered. After multiplying the design effect for cluster sampling (1.5) and adding 10% as the non-respondent rate, the sample size was 625. We selected 25 clusters, with 25 students from each cluster.

**Figure 1:**
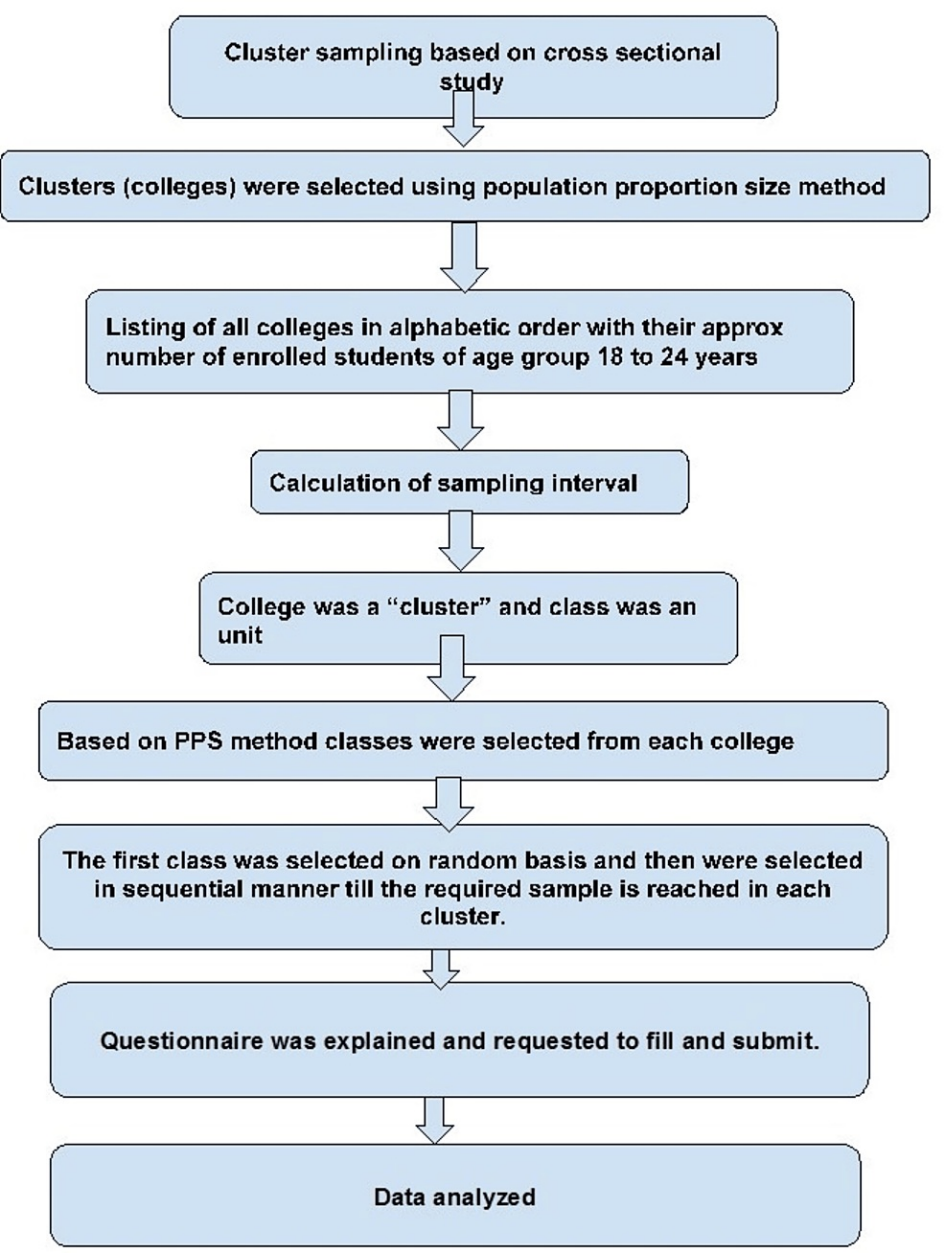
Flowchart for selection of study participants PSU=Primary Sampling Unit; SSU=Secondary Sampling Unit

Cluster sampling was done, where the college was the primary sampling unit and classes were the secondary sampling unit. A cluster was selected using the population proportion to size method (PPS). All the colleges with the number of approximate students were listed in alphabetical order. The sampling interval was calculated by dividing the required sample size by the cumulative total number of students in these colleges and rounding off to the nearest zero decimal. Individuals in class were a sampling unit. More than one class was selected in each college depending on the PPS method. The first class was selected randomly, and then participants were selected sequentially until the required sample was reached in each unit. If the number of students was less than required (i.e., 25), the next adjacent class was selected (Figure 1). Details of the sampling procedure are given in Annexure 1.

Study tool

We used a structured expert-validated questionnaire with four parts (A-D). Part A related to questions on sociodemographic profile, Part B is the composite lifestyle index tool for lifestyle domains - physical activity [21], dietary habits, stress, alcoholism, smoking, drug abuse, sweet [22], fruit and vegetable serving [23], Part C is the medical history, and Part D included questions related to knowledge and attitude regarding lifestyle.

Data collection procedure

Necessary administrative approvals from colleges were taken before the visit. The day and time of the visit for data collection were planned in consensus with the class teacher. The investigator carried all the necessary equipment (weighing machine, inch tape, required number of hard copies of the questionnaire to contour the possible Internet connectivity issue). After obtaining informed consent and explaining the questionnaire, students were asked to fill out the Google form or pen-paper-based form at their convenience. Only one response was accepted from each participant. After the survey, health education was given to the students about healthy lifestyle practices, their importance in the current era, and how the risk factors for NCDs can be prevented. The session ended by thanking the participants.

Statistical analysis

Data were entered in Microsoft Excel (Microsoft Corporation, Redmond, WA, US) and exported to IBM SPSS Statistics for Windows (Version 23.0. Armonk, NY: IBM Corp). Index values for each lifestyle activity were added to get a composite score. It ranged from 4 to 60, with 60 being a perfectly healthy lifestyle while 40 was the cut-off for a healthy lifestyle. Thomas L. Lenz gives details on composite lifestyle calculation [20].

In section D, two attitude-related questions were scored as five for strongly agree/very motivated and 1 for strongly disagree/not at all motivated. These two questions were averaged together, range 1 to 5. The attitude was categorized as 4-5 = favorable, 3 = neutral, and <=2 = unfavorable. Questions 3 and 11 were given three as the maximum score for knowledge scoring, whereas for the rest, a score of one was given for correct response. This was summed together, and a total of 11 questions with a score of 0-15 were categorized as good knowledge (>10), adequate knowledge (6-10), and poor knowledge (<6).

For association with clinical, social, and demographic factors, chi-square was used. Spearman correlation was used for the knowledge and attitude score with the lifestyle score. A P-value of less than 0.05 was considered statistically significant.

The study has been approved by AIIMS IEC (AIIMS/IEC/20/656). Confidentiality has been maintained throughout the manuscript.

## Results

Table 1 shows the sociodemographic profiles of the participants. A total of 742 students responded to the questionnaire, which was the sample size of the study. All questionnaires were filled out in the presence of the researcher. Only those who consented were given a questionnaire so all 742 consented and responded. There were approximately 25 students in each cluster. The majority of participants (55.12%) were in the age group 18-20 years, 61.99% were female, 59.43% of participants resided in their homes, and 46.78% of total participants belonged to the upper class followed by the upper middle.

**Table 1:** Sociodemographic characteristics of participants * Modified BG Prasad scale 2021 [24]

Sociodemographic factors			No. of participants (%)
Name of college (n=742)	College no 1	100 (13.50)	
	College no 2	36 (05)	
	College no 3	150 (20.21)	
	College no 4	109 (14.50)	
	College no 5	224 (31.96)	
	College no 6	40 (5.40)	
	College no 7	70 (9.43)	
Age in completed years (n=742)	18-20	409 (55.12)	
	20-22	254 (34.21)	
	22-24	79 (10.65)	
Gender (n=742)	Male	281 (37.90)	
	Female	460 (61.99)	
	Prefer not to say	1 (0.13)	
Residence (n=742)	Home	441 (59.43)	
	Hostel	220 (29.64)	
	Rental room	81 (10.93)	
Socioeconomic status * (n=742)	Upper class (7533 and above)	347 (46.78)	
	Upper middle class (3766 – 7532)	149 (20.08)	
	Middle class (2260 – 3765)	87 (11.72)	
	Lower middle (1130 – 2259)	104 (14.01)	
	Irrelevant income data	55 (7.41)	

**Figure 2:**
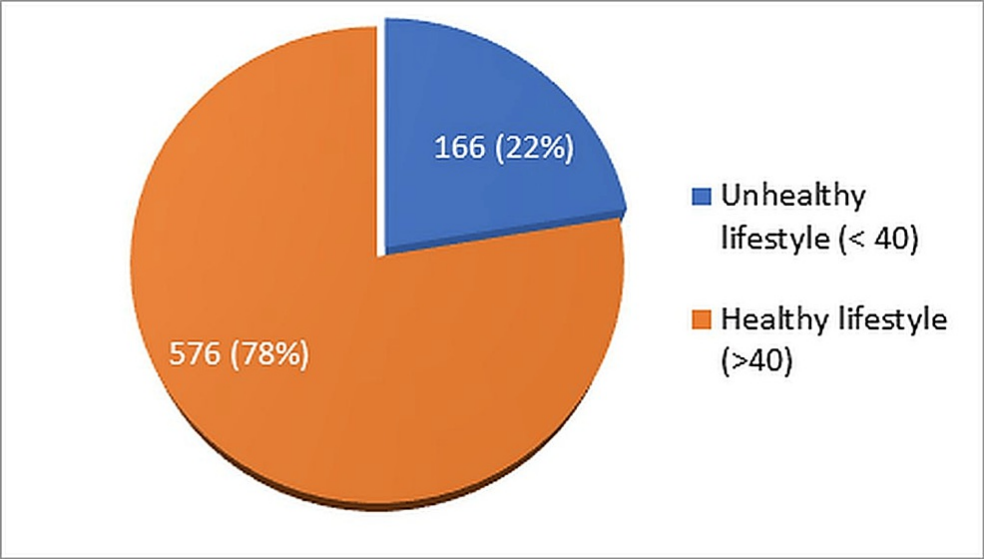
Prevalence of unhealthy lifestyle among study participants

Figure 2 depicts that 576 (77. 62%) participants of the study were following a healthy lifestyle with a composite lifestyle index (CLI) score of >40 while 166/742 participants (22.37 %) were living an unhealthy lifestyle with a score of < 40.

Table 2 shows that with progress in the professional year, the prevalence of unhealthy lifestyles also increases from 17.3% in the first year to 63.3% in the fourth professional year. Participants residing in hostel and rental rooms had a higher prevalence of unhealthy lifestyles compared to home dwellers. Medical, dental, and management students had a higher prevalence of unhealthy lifestyles compared to other streams. When a stream is further segregated by course, more than half the proportion of B.Sc nursing students (56%) were living unhealthy lifestyles.

**Table 2:** Sociodemographic characteristics and composite lifestyle index score of study participants i) B.Sc.: Bachelor of Science, ii) B.Ed.: Bachelor of Education, iii) D-Pharma: Diploma in Pharmacy, iv) B.Sc. Nurs.: Bachelor of Science in Nursing, v) B.Tech.: Bachelor of Technology. vi) B.Com: Bachelor of Commerce, vii) BBA: Bachelor of Business Administration, viii) BA: Bachelor of Arts, ix) MBBS: Bachelor of Medicine and Surgery, x) BDS: Bachelor of Dental Surgery, xi) CLI: composite lifestyle index *Modified BG Prasad scale 2021 [24]

Determinant			CLI < 40, n= 166 (22.3%)	CLI > 40, n= 576 (77.6%)	Total (%), 742 (100%)	Chi-square (p-value)
Gender		Male	53 (18.86%)	228 (81.14 %)	281 (100%)	3.26(0.07)
		Female	113 (24.56%)	347 (75.44 %)	460 (100%)	
		Not prefer to say	0 (0 %)	1 (100 %)	1 (100%)	
Age group		18- 20	80 (19.56%)	329 (80.44 %)	409 (100 %)	4.18 (0.12)
		20- 22	65 (25.60 %)	189 (74.40%)	254 (100 %)	
		22 - 24	21 (26.58 %)	58 (73.42%)	79 (100 %)	
Socioeconomic status *		Upper class	64 (18.44%)	283 (81.56%)	347 (100%)	9.32 (0.05)
		upper middle class	36 (24.16)	113 (75.84%)	149 (100%)	
		Middle class	17 (2.29%)	70 (9.43%)	87 (100%)	
		Lower middle class	10 (9.61%)	94 (90.39%)	104 (100%)	
		Erroneous income	39 (70.90%)	16 (29.10%)	55 (100%)	
Professional year		1st	45(17.30 %)	215 (82.69%)	260 (100 %)	20.3 (0.0001), 9.23 (0.0007)
		2nd	83(21.8%)	297 (78.15 %)	380 (100 %)	
		3rd	31(34.44%)	59 (65.55%)	90 (100 %)	
		4th	7 (58.34%)	5 (41.66%)	12 (100 %)	
		Stream	Medicine		33(32.03%)		70 (67.97%)	103 (100 %)
Dental			27(34.62%)	51 (65.38 %)	78 (100 %)	
Engineering			7(10.77%)	58 (89.23%)	65 (100 %)	
Science			61(19.24%)	256 (80.75%)	317 (100 %)	
Management			17(27.42%)	45 (72.58%)	62 (100 %)	
Art			11(20%)	44 (80%)	55 (100 %)	
Commerce			10(16.12%)	52 (83.88%)	62 (100 %)	
Residence	Home		70 (15.87%)	372 (84.35%)	442 (100%)		33.96 (< 0.001)
	Hostel		79 (35.90%)	141 (64.10%)	220 (100%)		
	Rental room		17 (21.25%)	63 (78.75%)	80 (100%)		
Course	B.Sc.		15 (5.12%)	155 (94.88%)	170 (100%)	66.7 (<0.001)
	B.Ed.		13 (28.89%)	31 (71.11%)	44 (100%)	
	D-Pharma		12 (17.91%)	55 (82.09%)	67 (100%)	
	B.Sc. Nurs.		21 (56.75%)	16 (43.24%)	37 (100%)	
	B.Tech.		7 (8.11%)	58 (91.89%)	37 (100%)	
	B.Com.		10 (17.46%)	52 (82.54%)	62 (100%)	
	BBA		17 (26.23%)	45 (73.78%)	62 (100%)	
	BA		11 (20%)	44 (80%)	55 (100%)	
	MBBS		33 (33%)	67 (67%)	100 (100%)	
	BDS		27 (34.62%)	51 (65.38%)	78 (100%)	

Table 3 depicts that 21.2% were underweight and 35.92 % were pre-obese or obese. Of those in the pre-obese group, 25.7% had an unhealthy lifestyle followed by normal weight (23.97%) and underweight (19%). Comparatively fewer obese people were found to have lower unhealthy lifestyles. This difference between BMI categories and CLI scores was not significant.

**Table 3:** BMI and composite lifestyle index score of study participants CLI: composite lifestyle index

BMI Group	CLI < 40	CLI > 40	Total	Chi-square p-value
Underweight	28 (19.31%)	117 (80.69%)	145 (100%)	3.5 (0.318)
Normal weight	70 (23.97%)	222 (76.03%)	292 (100%)	
Pre-obesity	27 (25.71%)	78 (74.29%)	105 (100%)	
Obesity class I	18 (17.82%)	83 (82.18%)	101 (100%)	
Obesity class II	7 (17.95%)	32 (82.05%)	39 (100%)	
TOTAL	150 (21.99%)	532 (78.01%)	682 (100%)	

Table 4 also depicts that the maximum number of students, i.e., 230, who had CLI > 40 scored 6-10, 184 scored ≥11, and 65 scored less than 5 in lifestyle-related knowledge assessment. The Pearson chi-square value showed that there was no significant relation between knowledge and the CLI group.

**Table 4:** Knowledge and attitude related to healthy lifestyle and composite lifestyle index score of study participants CLI: composite lifestyle index

Determinant		CLI < 40; n= 166 (22.3%)	CLI > 40; n= 576 (77.6%)	Total (%): 742 (100%)	Chi-square (p-value)
Knowledge score	≥11	59 (24.28)	184 (75.72)	243 (100)	0.4137 (0.81)
	6-10	65 (22.04)	230 (77.96)	295 (100)	
	≤ 5	17 (20.00)	65 (80.00)	81 (100)	
Attitude score	≥ 4	29 (14.95)	165 (85.05)	194 (100)	33.33 (<0.01)
	3	129 (24.06)	407(75.94)	536 (100)	
	≤ 2	8 (66.67)	4 (33.33)	12 (100)	

It also depicts that the majority of students, i.e., 407 participants with a CLI score >40 scored 3 followed by 165 participants with a CLI score >40, and 4 scored ≤2 in lifestyle-related attitude assessment. The Pearson chi-square value showed that there was a significant relation between attitude and CLI group.

Table 5 shows the logistic regression concerning unhealthy lifestyles and its important predictors. Naegellerker R2 was 0.174, i.e., this model explained 17. 4% of the variance. Course and attitude added significantly to the model. The odds of having an unhealthy CLI score (less than 40) is 11.9 times greater among B.Sc. Nursing students as compared to other BSc students. Every point increase in attitude score decreases the odds of a CLI score of less than 40 by 0.259 units (1-0.747). After adjusting, only attitude and course were found to be associated with CLI scores.

**Table 5:** Adjusted odds ratio for important variables of the study i) B.Sc.: Bachelor of Science, ii) B.Tech.: Bachelor of Technology, iii) B.Com.: Bachelor of Commerce, iv) MBBS: Bachelor of Medicine and Surgery, v) BDS: Bachelor of Dental Surgery

Variable	Log odds (LL-UL)	p-value
Female	Ref	0.87
Male	0.89 (0.574 - 1.386)	0.61
Home	Ref	0.31
Hostel	0.65 (0.91 – 4.699)	0.67
Rental room	1.65 (0.81 – 3.368)	0.16
Art	Ref	0.68
Commerce	.00 (0.000)	1.00
Dental	2.42 (.298 - 19.731)	0.47
Engineering	0.70 (.187 - 2.648)	0.64
Management	1.30 (.527 - 3.219)	0.56
Medical	2.78 (.330 - 23.457)	0.34
Science	0.64 (0.236 - 1.736)	0.38
First prof.	Ref	0.37
Second prof.	.85 (.511 - 1.419)	0.53
Third prof.	1.44 (.731 - 2.849)	0.29
Fourth prof.	1.25 (0.290 - 5.470)	0.75
BMI	0.96 (0.917 - 1.009)	0.10
Knowledge	0.96 (0.889 - 1.046)	0.37
Attitude	0.74 (0.594 - 0.939)	0.01
B.Sc.	Ref	0.03
B.Com.	32 (.000)	1.00
B.Sc. Nursing	11.09 (1.175 - 104.74)	0.03
B.Tech.	0.34 (.056 - 2.103)	0.24
MBBS	1.20 (.441 - 3.307)	0.71
BDS	1.28 (0.33 - 3.607)	0.78

## Discussion

The prevalence of unhealthy lifestyles among college-going adolescents in the current study was 22.37%, i.e., approximately one-fourth of the participants indulged in unhealthy lifestyle practices. Five factors, i.e., streams of the students, professional educational year, residence, attitude toward health, and chronic disease, were significantly associated with unhealthy lifestyles significant association was found between socioeconomic status and CLI score, representative study participants were from the middle or upper class only, with none from the lower class. The present study focused on unhealthy lifestyle patterns and health risk behaviors such as sweets, tobacco, drug and alcohol consumption, and obesity among college students. This study included a large sample size (n=742) of college-going adolescents who are more prone to unhealthy lifestyles. Anticipating the heterogeneity of adolescent groups studying in various colleges, cluster sampling was considered. We used the Composite Lifestyle Index score for scoring and assessing lifestyle behavior as lifestyle behaviors do not have one-to-one causation, but they interact with each other for the development of disease. We briefed students about the data collection tool for half an hour to get quality data through a self-administered questionnaire. We also educated them about healthy lifestyle practices.

Evangeline Mary A. et al., in 2017, surveyed 483 college students with pre-tested, semi-structured questionnaires based on UNICEF's 'Findings of the National Research on Adolescents' Attitude to Healthy Lifestyle and the WHO STEP-wise Approach Chronic Disease Risk Factor Survey' questionnaire. This study found that 78 % had reported an unhealthy lifestyle, and almost all had at least one behavioral risk factor [25]. Another study by Arun Pratap Singh et al. in three settings - Delhi, Lucknow, and Kaisarganj (UP) - reported 50-74% of unhealthy lifestyle behaviors among 1500 adolescents [26]. Joy Kumar Chakma et al. conducted the study among students of Delhi University (n=450) with the WHO STEP-wise approach and reported poor practices toward healthy lifestyles with a large number having NCD-related risk factors [27]. The prevalence of unhealthy lifestyles was much higher than in the current study, which may be due to it being based in metro cities and the current setting was the taluka level. The second reason may be variation in the questionnaire and self-reporting bias. We estimated a prevalence of approximately 50%, and the finding is lower than expected. The reason can be that most students, i.e., 59.43%, were residing at home with their parents/guardians due to COVID-19 lockdown restrictions. The present study was carried out during the phase-wise lifting of the COVID-19 restriction period. Higher education institutes were just opened for examination. The reason for the lower prevalence of unhealthy lifestyles in this study can be that most students, i.e., 59.43%, were residing at home with their parents/guardians due to COVID-19 lockdown restrictions. When students reside with their parents, under their guidance, they tend to be disciplined in terms of eating habits and sleep hours; they also tend to be less anxious. The institutes had just opened, and we asked them questions about the last four weeks (the reference period). Higher educational institutes were just opened for a certain period, movement was restricted, and the study was conducted during exam time. A comparative study to assess the health status and academic progress among day scholars and hostellers in nursing colleges of New Delhi reported that though most of the hostellers had good academic progress, day scholars had good health and healthy lives as compared with hostelers [28].

Students have more entertainment choices with computers and mobile phones, leading them to lose interest in outdoor activities and the heavy course load. Alzamil HA. et al. (2019) found that nearly half of the female students were physically inactive [29]. The current study also confirms that the prevalence of unhealthy lifestyles was higher in girls than boys. According to Regina Guthhold et al., in 2016, 27 countries had a prevalence of insufficient activity of 90% or more for girls compared to two countries for boys [8]. The prevalence of insufficient physical activity significantly decreased between 2001 and 2016 for boys and there was no significant change for girls. There was no clear pattern of physical activity according to country income group, similar to the present study showing no significant difference in the prevalence of unhealthy lifestyles between socio-economic groups.

Association of determinants of unhealthy lifestyle with CLI

We found a significant association of the CLI group with professional year, educational stream, type of residence, and attitude of participants toward health. In the present study, the prevalence of unhealthy lifestyles increased with the advancement in the professional year. It was highest in the fourth professional year (63.63 %), followed by the third year (34.44%). Germine El-Kassas found similar results [30]. Among those with CLI >40, i.e. healthy lifestyle, 34.54% were from science, and less than 10% were from the medicine and dental streams. Nursing students were found to have the highest prevalence of unhealthy lifestyle behavior. Contrary to our expectation, medicine, dental, and nursing students had higher unhealthy lifestyle behaviors than other streams, which may be due to stress, and many were hostellers. Hostellers had a statistically higher prevalence of unhealthy lifestyles (CLI <40) as compared to day scholars. In hostels, students are free to live and they tend to live unhealthily because of the absence of any guidance, and because of the absence of their near and dear ones, they could not get enough emotional support, which can lead them to stress, and demotivation. This can be the main reason for the higher prevalence of unhealthy lifestyles among hostelers in this age group in comparison to those of day scholars.

In the current study, no significant association was found between the knowledge score and the CLI score of the participants, which may be due to the limited sample size. We found that those with unfavorable attitudes toward health had lower CLI scores (<40) compared to their counterparts. After adjusting for other factors, attitude and nursing stream were significantly associated with the CLI score.

Considering the nature of data collection, there may be a response bias and in the respondents' tendency to answer in a socially desirable manner. We tried to reduce this bias by providing detailed information about the purpose of the study and assuring anonymity. We motivated the students to give truthful responses, which will help develop strategies for healthy practices in the college environment. As the study was limited to college-going, non-college-going adolescents weren't surveyed. Qualitative research methods can be utilized to analyze the reasons for unhealthy behaviors among adolescent students.

Limitations of this study

The present study was questionnaire-based so there may be a response bias and respondents' tendency to answer in a socially desirable manner. We tried to reduce this bias by giving detailed information about the study and anonymity before data collection. We motivated the students to give truthful responses, which will help develop strategies for healthy practices in the college environment. Another limitation was that we couldn’t survey the non-college-going adolescents. This study only analyzed the prevalence of unhealthy lifestyles quantitatively Qualitative research methods can be utilized in further studies to have an in-depth analysis of the reasons for unhealthy behaviors amongst adolescent students.

## Conclusions

The current study indicates that nearly a fourth of college students were having unhealthy lives during the COVID lockdown. Those studying in health streams were more likely to have unhealthy lifestyle behaviors than other streams. Those with unfavorable attitudes had a higher proportion of unhealthy lifestyle behaviors. It is recommended to start healthy lifestyle education early and sustain efforts throughout a child's school experience with the parents' and teachers' participation by creating a festive atmosphere and emphasizing that achieving and managing a healthy lifestyle can be fun. Students who are strongly motivated toward healthy eating and exercise should be given the lead role in influencing their peers. If unhealthy behavior is due to stress, boredom, or burnout, the same must be addressed appropriately.

The prevalence of unhealthy behavior was quite low in our study compared to others. We also concluded that, though the year 2020-2021 was rough due to the COVID-19 pandemic, it did teach us crucial lessons about health and lifestyle.

## Appendices

Annexure 1

**Table 6:** Cluster sampling procedure Sampling interval * = 143 College names are not given to maintain confidentiality.

Sr. no	Colleges in alphabetical order	Approx. students	Cumulative population	Random No. + SI	No. of clusters
1	College 1	525	525	114	3
				257	
				403	
2	College 2	270	795	549	2
				695	
3	College 3	700	1491	841	5
				987	
				1133	
				1279	
				1425	
4	College 4	663	2168	1571	4
				1717	
				1863	
				2009	
5	College 5	995	3166	2155	8
				2301	
				2447	
				2593	
				2739	
				2885	
				3031	
				3177	
6	College 6	150	3316	3323	1
7	College 7	260	3576	3469	2
				3615	
	Total	3576			
